# Perception of cultured “meat” by Italian, Portuguese and Spanish consumers

**DOI:** 10.3389/fnut.2023.1043618

**Published:** 2023-06-20

**Authors:** Jingjing Liu, João M. Almeida, Nicola Rampado, Begoña Panea, Élise Hocquette, Sghaier Chriki, Marie-Pierre Ellies-Oury, Jean-Francois Hocquette

**Affiliations:** ^1^INRAE, Université Clermont Auvergne, VetAgro Sup, UMR 1213, Recherches sur les Herbivores, Saint-Genès-Champanelle, France; ^2^INIAV, Quinta da Fonte Boa, Vale de Santarém, Portugal; ^3^Department of Agronomy, Food, Natural Resources, Animals and Environment (DAFNAE), University of Padua, Legnaro, Italy; ^4^Centro de Investigación y Tecnología Agroalimentaria de Aragón (CITA), Universidad de Zaragoza, Zaragoza, Spain; ^5^ISARA, Lyon, France; ^6^Bordeaux Sciences Agro, Gradignan, France

**Keywords:** survey, consumer, cultured “meat”, acceptance, barriers, motives

## Abstract

The aim of this study was to investigate how consumers (*n* = 2,171) originated from South-Western Europe (Italy, Portugal, and Spain) perceive cultured “meat” (CM) and if their demographic characteristics (origin, gender, age, education, occupation, and meat consumption) are related to their willingness to try (WTT), to regularly eat (WTE) and to pay (WTP) for CM. We found the current respondents had an initially positive attitude towards CM: 49% of them perceived CM as “promising and/or acceptable” and 23% “fun and/or intriguing” whereas 29% considered it as “absurd and/or disgusting”. In addition, 66 and 25% would be willing and not willing to try CM, respectively. However, 43% had no WTE for CM and, 94% would not pay more for CM compared to conventional meat. Age and especially occupation were good indicators of consumer acceptance of CM. Respondents of 18–30 years of age had the highest acceptance. Respondents outside the meat sector had the highest WTE and people working within the meat sector had the lowest WTE, scientists (within or outside the meat sector) had the highest WTT, people not scientists but within the meat sector had the lowest WTT. Additionally, we found that men are more likely to accept CM than women, Spanish-speaking consumers had the highest WTT and WTE, people with vegan and vegetarian diets may pay more for CM but generally no more than for conventional meat. The perceptions that CM may be more eco-friendly, ethical, safe and healthy than conventional meat, and to a lower extent, the perception that current meat production causes ethical and environmental problems are likely to be major motives for the current respondents to try, regularly eat and pay for CM. On the opposite, lower perceptions of CM benefits and of conventional meat weaknesses more generally, plus emotional resistance towards CM are main barriers to accept CM.

## Introduction

In recent years, the animal production industry has been challenged by the need to meet the growing global demand for meat while also reducing the negative impacts of livestock and meat production. These negative effects are highlighted in the press media (such as greenhouse gas emissions (GHG), feed-food competition, low animal welfare, and potential risks for human heath). Although meat consumption may be slowing down in developed countries, it is projected that global meat consumption will continue to grow as populations and incomes increase especially in some developing countries (1). As it is expected, global meat consumption would increase by 1.4% per year (2). Approximately 31% of global human-caused GHG emissions come from agri-food production, where meat accounts for a significant proportion (3). It is estimated that more than 70 billion farm animals are raised and slaughtered for meat production per year (4). A proportion of them is raised in intensive farming conditions and slaughtered at a very young age. Additionally, animal food consumption raises a set of health issues such as the risk of colorectal cancer (5, 6).

Dietary changes can bring ethical and environmental benefits to a large extent, which is not achieved yet by our food system (7). For most humans (except vegetarians), eating meat is perceived as natural, normal and necessary (8) with unique sensory and flavor properties. Meat consumption is reinforced by ingrained dietary habits, social norms, values and policy actions (7). Moreover, vegetarianism is a personal choice, meat-free diet may not be acceptable for everyone and may not be a solution anyway.

While some authors are promoting agroecology to change our current production systems to improve their sustainability (9, 10), the development of meat alternatives has been promoted as a good solution. Market share of meat substitutes grows as consumer acceptance increases due to potential ethical, safety and so-called environmental benefits. In fact, for example, plant-based meat alternatives generally may have less GHG than conventional meat (11). However, it depends on the production process, it was reported that in one scenario, plant-based meat production had higher carbon footprint than conventional beef produced on well-managed pastures (12).

Cultured “meat” (CM) is also presented to have a potential to drastically reduce environmental externalities according to some authors (13) but not all (14). As an innovation breakthrough in food production, the basic technique of CM is tissue engineering. Satellite muscle cells and fibro-adipogenic cells are cultured in culture medium allowing their proliferation and differentiation to produce muscle fibers and fat tissues which are presented as meat when mixed together (15). Even if the production of animal-free medium seems still uncertain in terms of large scale production and cost (16), and even the biopsy procedure can still cause animal pain to some extent, the introduction of CM is likely to reduce the number of slaughtered animals. Although the process of culturing cells is hardly ever perfectly controlled and even if some unexpected safety issues may arise (17), food-borne pathogens is likely to be reduced compared to animal farming and production. As for the potential environmental benefits CM can bring, it has so far not been precisely assessed but relies more on forecasts. It will take a long time to confirm these conclusions, which makes them highly speculated (18). As it was demonstrated by Escobar et al. (14), the calculation of the beneficial contribution of CM to the environment depends on how much reliable data is available.

Compared to other meat alternatives, CM might be the closest one to meat in terms of molecular and organoleptic properties even its composition is not known yet (19). Despite so, CM is less favored than plant- and even insect-based meat substitutes (20–22). Moreover, this may incite vegetarians who like meat but do not eat meat for animal and environment protection to eat meat again. Although it was found that vegetarians would be more likely to perceive the potential benefits of CM, they were still less interested in trying it (23).

At present, CM is not yet commercially available, except for the first commercial product (chicken bites/chicken nuggets from the American startup Eat Just) distributed in 1,880 restaurants of the same chain in Singapore after receiving approval from the Singapore Food Authority (24). Thus, the commercial availability of the first cultured meat (CM) product lays the foundation and paves the way for other such meat alternatives to enter the worldwide food market (25). Without actually being exposed to the product, the study of consumer acceptance of CM is based on hypothesis and assumptions. To date, most of the literature has examined consumer acceptance through survey studies. Although more and more studies have been conducted with representative samples, what the population would be willing to do is not precisely known since consumers’ answers vary according to origin and cultural factors. However, from a practical point of view, hypothetical studies on the acceptance and/or willingness of a representative population for a product that is not yet available (such as CM) may be inherently less reliable. Herein, the study of consumer acceptance would be more relevant if we could find some indicators (i.e., motives, barriers) that would be good predictors of consumer acceptance.

The main factors that are affecting consumer perception and acceptance of CM are ethical, environmental concerns and issues related to the production process, in interaction with doubts, neophobia, fear and disgust (26). The perception of those factors varies between consumers in different sociodemographic segments. This study therefore seeks to obtain data which would enrich consumer acceptance research on CM. There are two primary aims of this study: (1) to capture perception of CM in specific consumer segments from citizens with origin from South-Western Europe, and (2) to investigate potential indicators (motives, barriers) of consumer acceptance of CM. Results will be analyzed considering published data with respondents from other countries (China, Brazil, France) using the same survey (27–29). Furthermore, compared to our previous studies, novel statistical approaches were developed to better identify barriers and motives of acceptance of CM.

## Materials and methods

### Questionnaire design

As mentioned above, this study is an extension in other countries of previous published research with the same questionnaire (27–29) and with additional statistical approaches. Only some of the questions below and data were used in order to obtain key information that met the purpose of this study. The complete questionnaire can be seen in Hocquette et al. (29). The questions used in this study are presented in Table 1.

**TABLE 1:** Questions of the CM questionnaire used in this study.

Section	Question	Answer/Scale
Demographic information	Gender	Female; Male; No answer
	Age	18–30 years of age; 31–50 years of age; >50 years of age
	Origin	Italian - people originated from Italy and/or mainly Italian-speaking; Portuguese - people originated from Portugal and/or mainly Portuguese-speaking; Spanish - people originated from Spain and/or mainly Spanish-speaking, even if few respondents live in South America.
	Education	Lower education - prior to university; Medium education - studying at university or having obtained a bachelor’s degree; Higher education - having obtained a master’s degree (or studying to do so) and above.
	Income (monthly net income)	0–1,500 €; 1,500–2,000 €; 2,000–2,500 €; 2,500–3,000 €; 3,000–4,000 €; >4,000 €; No answer Income level was sorted as: Low income – <2,000 €; Medium income – 2,000−3,000 €; High income –>3,000 €
	Occupation	Not scientists, work outside the meat sector; Not scientists, work within the meat sector; Scientists, work outside the meat sector; Scientists, work within the meat sector.
	Meat consumption	Never: vegetarian or vegan diet; Rarely: weekly or less; Regularly: several times a week; Daily or at every meal
	Familiarity with CM	Yes, I have heard of CM; No, I never heard of CM
Societal challenges that faced by conventional meat production	Do you think the conventional meat industry cause ethical problems?	(1)-Much less; (2)-Less; (3)-Unsure; (4)-More; (5)-Much more
	Do you think the conventional meat industry cause environmental problems?	(1)-Much less; (2)-Less; (3)-Unsure; (4)-More; (5)-Much more
	Do you think reducing meat consumption could be a good solution to resolve above problems?	(1)-Much less; (2)-Less; (3)-Unsure; (4)-More; (5)-Much more
Potential challenges that faced by CM	How ethical do you think CM would be compared to conventional meat?	(1)-Much less; (2)-Less; (3)-Unsure; (4)-More; (5)-Much more
	How eco-friendly do you think CM would be compared to conventional meat?	(1)-Much less; (2)-Less; (3)-Unsure; (4)-More; (5)-Much more
	How healthy, safe and nutritious do you think CM would be compared to conventional meat?	(1)-Much less; (2)-Less; (3)-Unsure; (4)-More; (5)-Much more
	How tasty do you think CM would be compared to conventional meat?	(1)-Much less; (2)-Less; (3)-Unsure; (4)-More; (5)-Much more
Acceptance of CM	Would you be willing to try CM?	(1)-Definitely no; (2)-Probably no; (3)-Unsure; (4)-Probably yes; (5)-Definitely yes
	Would you be willing to eat regularly CM?	0-No (I do not want to eat CM regularly); 1-Yes (I want to eat CM at restaurant/home/in ready-to-eat meals)
	How much would you be willing to pay for CM compared to conventional meat?	(1)-Much less than conventional meat; (2)-Less than conventional meat; (3)-Same as conventional meat; (4)-More than conventional meat; (5)-Much more than conventional meat
Perception of CM	What do you think of CM?	It is promising and/or acceptable; It is fun and/or intriguing; It is absurd and/or disgusting
	Do you have emotional resistance to accept CM?	(1)-Much less; (2)-Less; (3)-Unsure; (4)-More; (5)-Much more

### Data collection

The survey was distributed in Italy, Spain and Portugal through social networks and on campus questionnaire dissemination. Although, we note that a small part of the data was collected from people who live in other countries but speak Italian, Spanish and Portuguese, these data was still worthwhile to be used since either people originated from these three countries or those who speak these three languages as their first language are considered to have the corresponding cultural, local and dietary backgrounds. In the end, 2,171 answers including 46.7% Italian data, 31% Portuguese data and 22.3% Spanish data were collected, and the demographic information is detailed in Tables 1, 2.

**TABLE 2:** Demographic information of the current respondents (*n* = 2,171).

Demographic	Category	Number	Percentage
Gender	Female	1,232	56.7
	Male	923	42.5
Age	18–30 years of age	807	37.2
	31–50 years of age	857	39.5
	>50 years of age	507	23.4
Origin	Italy	1,014	46.7
	Portugal	673	31.0
	Spain	484	22.3
Education	Low level	42	1.9
	Medium level	1,144	52.7
	High level	960	44.2
Occupation	Not scientists and outside the meat sector	1,025	47.2
	Not scientists and within the meat sector	321	14.8
	Scientists outside the meat sector	555	25.6
	Scientists within the meat sector	270	12.4
Monthly net income	0–1,500 €	782	36.0
	1,500–2,000 €	386	17.8
	2,000–2,500 €	219	10.1
	2,500–3,000 €	98	4.5
	3,000–4,000 €	76	3.5
	>4,000 €	89	4.1
	No answer	521	24.0
Income*	Low income	1,168	53.8
	Medium income	317	14.6
	High income	165	7.6
	No answer	521	24.0
Meat consumption	Never: vegetarian or vegan diet	214	9.9
	Rarely: weekly or less	477	22.0
	Regularly: several times a week	1,268	58.4
	Daily or at every meal	212	9.8
Familiarity	Ever heard	1,660	76.5
	Never heard	511	23.5

*Classification of monthly net income into different levels.

### Statistical analysis

The data was analyzed using R software (version. 4.1.1) and IBM SPSS 25 depending on the different output needs such as plotting.

The demographic variables and their categories are presented in Tables 1, 2. Some treatments of categories need to be specified such as for age, 18–30 years of age was considered as young, 31–50 years of age was considered as middle-aged and more than 51 years of age was considered as senior or old in this study. For meat consumption, people never eat meat were considered as vegans and/or vegetarians. These treatments were used in the following analysis with General Linear Model (GLM) and logistic regression modeling. Three types of statistical analyses were performed with this transformed data set.

First, variance analysis (ANOVA) was performed with the GLM procedure in SPSS as previously described (27, 29) to examine the difference in WTT, WTE, and WTP depending on respondent groups based on different demographic characteristics. As in previous studies (27), some, but not all of the assumptions of ANOVA were sometimes violated in this survey case, such as normality of distributions and homogeneity of variances. Therefore, we ran a Welch’s ANOVA, which does not require the homogeneity of variance assumption, and we obtained extremely similar results compared to ANOVA which is considered as being robust (30). Based on these observations, we proceeded with ANOVA since the Welch’s ANOVA does not accept interactions. *Post-hoc* test was performed using Bonferroni test with the Bonferroni correction for pairwise comparisons between groups with significant difference, which was determined at the level of *p* < 0.05.

Second, to identify the potential motives and barriers to the acceptance of CM, Pearson correlation analysis was thereafter performed by R software to determine the relationships between variables (WTT, WTE, WTP, and other questions regarding the perception of conventional meat production and of CM). To have an overall perception of conventional meat production and CM, respectively, two overall variables “Overall perception of conventional meat production” and “Overall perception of CM” were created. Overall perception of conventional meat production was calculated by merging answers to two questions: (1) Does meat production cause ethical problems? and (2) Does meat production cause environmental problems? as follow 0.5 × answer to question 1 + 0.5 × answer to question 2 (both from a scale from 1 to 5). Overall perception of CM was calculated by merging answers to four questions: (1) How ethical do you think CM would be compared to conventional meat? (2) How eco-friendly do you think CM would be compared to conventional meat? (3) How healthy, safe and nutritional do you think CM would be compared to conventional meat? and (4) How tasty do you think CM would be compared to conventional meat? as follow 0.25 × answer to question 1 + 0.25 × answer to question 2 + 0.25 × answer to question 3 + 0.25 × answer to question 4.

Third, logistic regression was developed to identify barriers and motives to accept CM. As demonstrated by Verbeke et al. (31), since this novel product is not yet commercially available on a large scale and to be consumed frequently, it is difficult to obtain real data regarding consumer WTT and WTE. Nevertheless, consumer willingness is highly driven by the perception of the product, and emotional resistance to the concept of CM can negatively affect consumers’ perception of this product and their willingness to try and eat (27, 29). Combined with relevant demographic variables, this analysis considered also the variability in emotional resistance, allowing to investigate the potential profile of CM adopters and rejectors. This modeling approach was greatly inspired by the research of Verbeke et al. (31).

Willingness to try for CM was analyzed as a discrete choice (yes-1, no-0) by combining the response categories “definitely yes” and “probably yes” as “yes” (65.5%), and “definitely no” and “probably no” as “no” for WTT (24.7%), “Unsure” was not used in this analysis; “I would be willing to regularly eat CM at restaurant/home/in ready-to-eat meals” as “yes” (56.7%) and “I do not want to regularly eat CM” as “no” (43.3%).

Binary logistic regression was used to model the discrete choice in terms of WTT and WTE. If the latent variable z*_*i*_* is greater than zero, the binary response y*_*i*_* (for WTT or WTE) for respondent *i* takes a value of one; otherwise, y*_*i*_* takes a value of zero:


$$\left\{ \begin{array}{c} y_{i}=1\,i\,f\,z_{i}>0\\ y_{i}=0\,i\,f\,z_{i}\leq 0 \end{array}\right.$$

The latent variable z*_*i*_* is constructed with a regression model where x*_*ki*_* represents explanatory variables that from 1 to *k* explaining WTT and WTE for participant *i* with β*_*k*_* as the coefficient that indicates the effect of *x*_*ki*_ on *z*_*i*_, and where ε*_*i*_* represents the random error for respondent *i*, as below:


$$z_{i}=\beta_{0}+\sum_{k=1}^{k}\left(\beta_{k}\,x_{k\,i}\right)+\varepsilon_{i}$$

In this study, z*_*i*_* is specified by a set of explanatory variables as *z_*i*_* = β*0* + β*1*Gender[Female]*_*i*_* + β*2*Age[> 50 years of age]*_*i*_* + β*3*Origin[Italy]*_*i*_* + β*4Occupation*[not scientists working within the meat sector]*_*i*_* + β*5*Meat consumption [Never]*_*i*_* + β*6*Income[high income]*_*i*_* + β*7*Education[high level]*_*i*_* + β*8*Familiarity[never heard]*_*i*_* + ε*i*.

In the current model, females, more than 50 years of age, originated from Italy, not scientist, working in the meat sector, who never consume meat (considered as vegans/vegetarians), with a high income, a high education level and who never heard about CM served as the reference category for demographic variables of gender, age, origin, occupation, meat consumption, income, education and familiarity with CM.

The logistic function used to transform *y*_*i*_ from *z*_*i*_ is based on the relationship between the probability *p*_*i*_ of dependent variable *y*_*i*_ (WTT or WTE) and the explanatory variable *x*_*k*_ (gender, age, etc.) as below:


$$p_{i}=prob\left(y_{i}=1\right)=\frac{e^{z_{i}}}{1+e^{z_{i}}}=\frac{e^{\beta_{0}}+\sum_{k=1}^{k}\beta_{k}\,x_{k\,i}}{1+e^{\beta_{0}}+\sum_{k=1}^{k}\beta_{k}\,x_{k\,i}}$$

Meanwhile:


$$l\,o\,g\,\left(\frac{p_{i}}{1-p_{i}}\right)=z_{i}=\beta_{0}+\sum_{k=1}^{k}\left(\beta_{k}\,x_{k\,i}\right)+\varepsilon_{i}$$

regression coefficient (β) was estimated based on maximum likelihood estimation and is presented with odds ratio [EXP (β), OR] and significance level (*p*-value).

## Results

### Characteristics and overall answers of the respondents

#### Basic demographic information of the current respondents

Of the total respondents, 56.7% were females and 42.5% were males (and 0.8% were unwilling to answer this question), of whom 46.7% were originated from Italy and/or mainly Italian-speaking, 31% were originated from Portugal and/or mainly Portuguese-speaking and 22.3% were originated from Spain and/or mainly Spanish-speaking. The current sample was mainly middle-aged and young people (39.5 and 37.2%, respectively), more than half (53.8%) had a net monthly income of less than 2,000 €. Most of them were well-educated (98.1% pursuing and/or have obtained a degree of bachelor, master or Ph.D.), working outside the meat sector (72.8%), being meat eaters (90.1%) with various frequencies (from rarely to daily), and being familiar with CM or at least have heard about this novel food biotechnology (Table 2).

#### Overall perception and willingness

Considering together all answers from the current respondents, the overall perception of conventional meat production and CM were observed as well as their WTT, WTE, and WTP CM (Table 3). In general, more than half of the current respondents believe that conventional meat production does cause a considerable amount of ethical and environmental problems (54.3, 62.6%) and reducing meat consumption could be a good solution to resolve those problems for 50.7% of the respondents. In general, they do believe CM would be more ethical and eco-friendly than conventional meat, but do not seem to be too much convinced that CM could be safer and tastier than conventional meat. This novel biotechnology does not provoke much emotional resistance (only of 32.8% of the current participants). In addition, 48.8% of them considered CM as “promising and/or acceptable,” 22.7% perceived CM as “fun and/or intriguing” and 28.5% felt CM as “absurd and/or disgusting.” Overall, the current respondents would be willing to try and regularly eat CM, but would be only willing to pay a lower price than conventional meat. A total of 65.5% of the respondents would be willing to try CM (26.7% answered “Definitely yes” and 38.8% “Probably yes”), 34.5% respondents were unwilling to try or were unsure about CM (10.5% answered “Definitely no,” 14.2% “Probably no” and 9.8% “Unsure”). A total of 56.7% of respondents would be willing to regularly eat CM (at home, restaurants or in ready-made meals), which means that 43.3% of respondents did not want to regularly eat CM at all. Only 5.7% of respondents would be willing to pay more for CM than conventional meat, 31.5% would be willing to pay the same price for CM as conventional meat, 62.8% would be willing to pay only less or much less or even nothing.

**TABLE 3:** Current perceptions of conventional meat production and cultured “meat” (CM) and willingness to try (WTT), willingness to eat (WTE), willingness to pay (WTP) for cultured “meat” (CM) based on 2,171 responses.

Perception of conventional meat production^1^	Mean	SD
Do you think the conventional meat industry cause ethical problems?	3.55	1.30
Do you think the conventional meat industry cause environmental problems?	3.73	1.28
Do you think reducing meat consumption could be a good solution to resolve above problems?	3.38	1.45
**Perception of CM** ^1^		
How ethical do you think CM would be compared to conventional meat?	3.07	1.42
How eco-friendly do you think CM would be compared to conventional meat?	3.09	1.35
How healthy, safe and nutritional do you think CM would be compared to conventional meat?	2.85	1.24
How tasty do you think CM would be compared to conventional meat?	2.46	1.21
Do you have emotional resistance to try CM?	2.78	1.44
**Willingness**		
Would you be willing to try CM?^1^	3.57	1.30
Would you be willing to regularly eat CM?^2^	0.57	0.50
How much would you be willing to pay for CM compared to conventional meat?^3^	2.22	0.87

^1^Response rated as (1)-Much less, (2)-Less, (3)-Unsure, (4)-More, (5)-Much more or Response rated as (1)-Definitely no, (2)-Probably no, (3)-Unsure, (4)-Probably yes, (5)-Definitely yes.

^2^Response rated as 0-No, 1-Yes.

^3^Response rated as (1)-Much less than conventional meat, (2)-Less than conventional meat, (3)-Same as conventional meat, (4)-More than conventional meat, (5)-Much more than conventional meat.

### Determinants of WTT, WTE, and WTP for CM

#### Determinants of WTT

A logistic regression was performed to ascertain the effects of gender, age, origin, occupation, meat consumption, income, education and familiarity on the likelihood that participants would be willing to try and to regularly eat CM. The model correctly classified 72.6% of the current responses of WTT. No significant differences were found for WTT between males and females (*p* > 0.05, Table 4) based on variance analysis in general linear model; nevertheless, the odds ratio of WTT is 1.3 times (OR = 1.29) greater for males as opposed to females (*p* < 0.05, Table 5) in logistic regression model. There was no difference of WTT between mid-aged and old participants (*p* > 0.05, Tables 4, 5). Spanish-speaking participants had the highest WTT (*p* < 0.001, Table 4) with 2 times (OR = 2.18) more likely than Italian-speaking participants, Portuguese-speaking people were 1.7 times (OR = 1.66) more likely to try CM than Italian-speaking participants (*p* < 0.001, Table 5). Low educated participants had the lowest WTT than medium and high educated people (*p* < 0.01, Table 4). There was no difference for WTT between different income groups (*p* > 0.05, Table 4), also, the predictive effect of income to WTT was not significant in the current logistic regression model (*p* > 0.05, Table 5). Scientists outside the meat sector had the highest WTT (*p* < 0.001, Table 4) and were 4 times (OR = 3.96) more likely to try CM than people who were not scientists but work within the meat sector that had the lowest WTT (*p* < 0.001, Table 5). Participants who were meat scientists were 2.7 times (OR = 2.66) more likely to try CM than participants who were not scientists but work within the meat sector (*p* < 0.001, Table 5). Participants who were not scientist and outside the meat sector were 2.4 times (OR = 2.41) more likely to try and eat CM than people who were not scientists but work within the meat sector (*p* < 0.001, Table 5). There was no difference for WTT among groups with different meat consumption levels (*p* > 0.05, Table 4). Nonetheless, rarely meat eaters were almost two times (OR = 1.80) more likely to try CM than vegans/vegetarians (*p* < 0.01, Table 5).

**TABLE 4:** Respondents’ willingness to try (WTT), willingness to eat (WTE) and willingness to pay (WTP) for cultured “meat” (CM) according to demographic categories.

Demographic	Category	WTT^1^		WTE^2^		WTP^3^	
		Mean	SD	Mean	SD	Mean	SD
Gender	Female	3.53	1.27	0.56	0.50	2.32^a^	0.85
	Male	3.63	1.33	0.58	0.49	2.09^b^	0.88
Age	18–30 years of age	3.80^a^	1.24	0.65^a^	0.48	2.40^a^	0.87
	31–50 years of age	3.47^b^	1.31	0.54^b^	0.50	2.16^b^	0.85
	>50 years of age	3.39^b^	1.33	0.49^b^	0.50	2.05^b^	0.85
Origin	Italy	3.55^b^	1.38	0.53^b^	0.50	2.32^a^	0.92
	Portugal	3.47^b^	1.22	0.54^b^	0.50	2.09^b^	0.84
	Spain	3.75^a^	1.22	0.68^a^	0.57	2.19^b^	0.87
Education	Low level	3.05^b^	1.23	0.40	0.50	1.98	0.75
	Medium level	3.53^a^	1.34	0.57	0.50	2.24	0.90
	High level	3.65^a^	1.25	0.57	0.50	2.21	0.83
Occupation	Not scientists outside MS^4^	3.53^b^	1.28	0.61^a^	0.49	2.30^a^	0.86
	Not scientists within MS	3.08^c^	1.40	0.39^b^	0.49	1.79^c^	0.84
	Scientists outside MS	3.86^a^	1.21	0.65^a^	0.48	2.39^a^	0.84
	Scientists within MS	3.70^ab^	1.28	0.46^b^	0.50	2.09^b^	0.82
Income	Low income	3.59	1.29	0.58^a^	0.49	2.22^ab^	0.84
	Medium income	3.58	1.32	0.49^b^	0.50	2.13^b^	0.88
	High income	3.59	1.41	0.53^ab^	0.50	2.12^ab^	0.95
	No answer	3.52	1.27	0.59^a^	0.49	2.30^a^	0.90
Meat consumption	Never	3.46	1.46	0.56	0.50	2.94^a^	0.85
	Rarely	3.69	1.26	0.60	0.49	2.31^b^	0.84
	Regularly	3.55	1.28	0.56	0.50	2.09^c^	0.80
	Daily	3.52	1.36	0.56	0.50	2.08^c^	0.98
Familiarity	Ever heard	3.61a	1.33	0.55^b^	0.48	2.17	0.79
	Never heard	3.44b	1.21	0.62^a^	0.50	2.23	0.89

^1^Response rated as (1)-Definitely no, (2)-Probably no, (3)-Unsure, (4)-Probably yes, (5)-Definitely yes.

^2^Response rated as 0-No, 1-Yes.

^3^Response rated as (1)-Much less than conventional meat, (2)-Less than conventional meat, (3)-Same as conventional meat, (4)-More than conventional meat, (5)-Much more than conventional meat.

^4^MS, meat sector. Within each demographic category, mean values with different superscript letters significantly differ from each other at the level of p < 0.05.

**TABLE 5:** Binary logistic regression explaining odds ratio (OR) of respondents’ willingness to try (WTT) and willingness to eat (WTE) cultured “meat” (CM) according to sociodemographic characteristics.

Category (ref)		WTT^1^			WTE^2^		
		β^3^	OR^4^	*P*-value	β	OR	*P*-value
Gender (Female)	Male	0.25	1.29	<0.05	0.34	1.27	<0.05
Age (>50 years of age)	18–30 years of age	0.81	2.24	<0.001	0.71	2.04	<0.001
	31–50 years of age	0.23	1.26	0.093	0.16	1.17	0.196
Origin (Italy)	Spain	0.78	2.18	<0.001	1.07	2.92	<0.001
	Portugal	0.51	1.66	<0.001	0.56	1.75	<0.001
Occupation (Not scientists working within MS)	Scientist working within MS^5^	0.97	2.66	<0.001	0.47	1.60	<0.01
	Scientist working outside MS	1.38	3.96	<0.001	1.31	3.69	<0.001
	Not scientist working outside MS	0.88	2.41	<0.001	0.99	2.68	<0.001
Meat consumption (Never)	Daily	0.20	1.22	0.427	–0.05	0.96	0.838
	Regularly	0.30	1.35	0.117	–0.01	0.99	0.997
	Rarely	0.59	1.80	<0.01	0.35	1.41	0.053
Income (High income)	Low income	–0.09	0.91	0.661	0.05	1.05	0.798
	Medium income	0.007	1.01	0.997	–0.15	0.86	0.460
Education (High level)	Low level	–2.81	0.76	0.362	0.19	1.02	0.946
	Medium level	0.11	1.11	0.455	0.33	1.39	0.007
Familiarity (Never heard)	Heard before	0.18	1.20	0.169	–0.17	0.85	0.143

^1^Willingness to try (WTT), the score of WTT was converted into a binary score, the scores of 1 and 2 were converted into 0 (unwilling to try), the score of 4 and 5 were converted into 1 (would be willing to try).

^2^Willingness to eat (WTE), 0–unwilling to regularly eat, 1–would be willing to regularly eat.

^3^β, regression coefficient β is associated with the expected change in log odds of dependent variable (WTT or WTE) per unit change in the explanatory variable.

^4^OR, odds ratio represents the constant effect of an explanatory variable, on the likelihood that dependent variable will change (WTT or WTE).

^5^MS, meat sector.

#### Determinants of WTE regularly CM

The logistic regression model correctly classified 63.2% of the current responses of WTE. According to ANOVA, no significant differences were found for WTE for CM between males and females (*p* > 0.05, Table 4) but according to logistic regression model, the odds ratio of WTE is 1.3 times (OR = 1.27) greater for males as opposed to females (*p* < 0.05, Table 3). Young people had the highest WTE (*p* < 0.001, Table 4) because they were twice (OR = 2.04) more likely than mid-aged and old people to regularly eat CM, there was no difference of WTE between mid-aged and old participants (*p* > 0.05, Tables 4, 5). Spanish-speaking people had the highest WTE (*p* < 0.001, Table 4) because they were three times (OR = 2.92) more likely than Italian-speaking people to regularly eat CM (*p* < 0.001, Table 5), Portuguese-speaking people were 1.8 times (OR = 1.75) more likely to eat CM than Italian-speaking people (*p* < 0.001, Table 5). Scientists outside the meat sector had the highest WTE (*p* < 0.001, Table 4) and were 3.7 times (OR = 3.69) more likely to eat CM than people who were not scientists but work within the meat sector that had the lowest WTE (*p* < 0.001, Table 4). Participants who were meat scientists were 1.6 times more likely to eat CM than people who were not scientists but work within the meat sector, people who were not scientists and outside the meat sector were 2.7 times (OR = 2.68) more likely to eat CM than people who were not scientists but work within the meat sector (*p* < 0.001, Table 5). Participants with the lowest income had higher WTE than medium income people (*p* < 0.05, Table 4). There was no difference for WTE among groups with different meat consumption levels (*p* > 0.05, Table 4). Participants who had heard about CM had higher WTT (1.2 times higher than people who had never heard about it) but lower WTE (*p* < 0.01, Table 4).

#### WTP for CM

Since only 5.7% of the current respondents would be willing to pay for CM at a price higher than conventional meat, it is difficult to transform the data with five categories in binary responses and this makes it impossible to apply logistic regression with WTP. Based on variance analysis with general linear model, it can be seen that females, young people and Italian-speaking participants of the current respondent sample were willing to pay more than males, middle-aged and old people, and compared to Spanish- and Portuguese-speaking participants (*p* < 0.001, Table 4). Participants working outside the meat sector would be willing to pay the most and participants working within the meat sector especially those who were not scientists would pay the least for CM (*p* < 0.001, Table 4).

### Motives and barriers of CM acceptance

#### Relationships between participants’ acceptance and perception of conventional meat production and of CM

**FIGURE 1:**
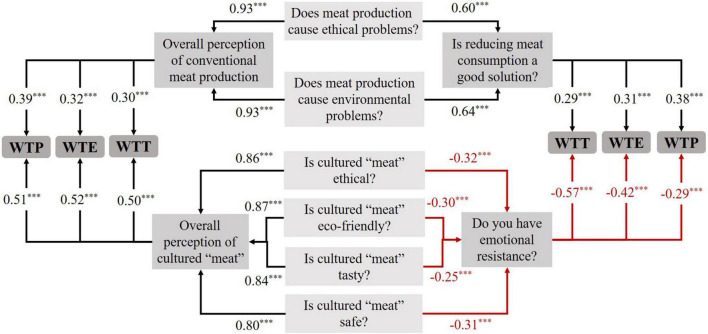
Correlation analysis between willingness to try (WTT), willingness to eat (WTE), willingness to pay (WTP) and overall perception of conventional meat production and cultured “meat” (CM). To have an overall perception of conventional meat production and CM, respectively, two overall variables “Overall perception of conventional meat production” and “Overall perception of CM” were created. Overall perception of conventional meat production was calculated by merging answers to two questions: (1) Does meat production cause ethical problems? and (2) Does meat production cause environmental problems? as follow (0.5 × answer to question 1 + 0.5 × answer to question 2) (both from a scale from 1 to 5). Overall perception of CM was calculated by merging answers to four questions: (1) How ethical do you think CM would be compared to conventional meat? (2) How eco-friendly do you think CM would be compared to conventional meat? (3) How healthy, safe and nutritional do you think CM would be compared to conventional meat? and (4) How tasty do you think CM would be compared to conventional meat? as follow (0.25 × answer to question 1 + 0.25 × answer to question 2 + 0.25 × answer to question 3 + 0.25 × answer to question 4). Positive correlations are presented in black, negative correlations are presented in red. ^***^Means that the correlation is significant at *p* < 0.001 level.

Figure 1 illustrates the correlations between participants’ willingness to try, to regularly eat and to pay for CM and their overall perception of conventional meat and of CM.

The perception of ethical and environmental problems caused by conventional meat production has positive correlations with the wish of consumers to reduce their meat consumption (*r* = 0.60, 0.64, *p* < 0.001). Considering that reducing meat consumption is a good solution is positively correlated with WTT, WTE, and WTP for CM (*r* = 0.29, 0.31, 0.38, *p* < 0.001). On the opposite, emotional resistance about CM is negatively correlated with the perception that CM may be ethical, eco-friendly, tasty and safe (*r* = -0.32, -0.30, -0.25, -0.31, *p* < 0.001). Emotional resistance is also negatively correlated with WTT, WTE, and WTP (*r* = -0.57, -0.42, -0.29, *p* < 0.001).

The overall negative perception of conventional meat production is positively correlated with WTT, WTE, and WTP (*r* = 0.30–0.39, *p* < 0.001). Similarly, the overall positive perception of CM (perceived as ethical, eco-friendly, tasty, and safe) is positively correlated with WTT, WTE, and WTP (*r* = 0.50–0.52, *p* < 0.001).

#### Predicted probabilities for WTT and WTE CM

Wald Chi-Square (χ^2^) value in logistic regression models indicates the predictive power of explanatory variables to the dependent variable. The Wald χ^2^ of WTT for gender, age, origin, occupation, meat consumption level, income, education and familiarity are 6.90, 28.66, 20.91, 60.67, 9.66, 1.37, 2.03, and 1.89. The Wald χ^2^ of WTE for gender, age, origin, occupation, meat consumption, income, education and familiarity are 8.87, 31.9, 48.9, 82.1, 9.28, 1.94, 7.77, and 2.15. Age, origin and occupation are therefore the most influential factors in both models of WTT and WTE. Considering the effect of origin can be partly skewed due to different sample sizes (46.7, 31.0, and 22.3% of Italian-, Spanish- and Portuguese-speaking participants) and due to nested effects with gender, age and other factors, only the effects of age and occupation are analyzed further in interaction with emotional resistance.

**FIGURE 2:**
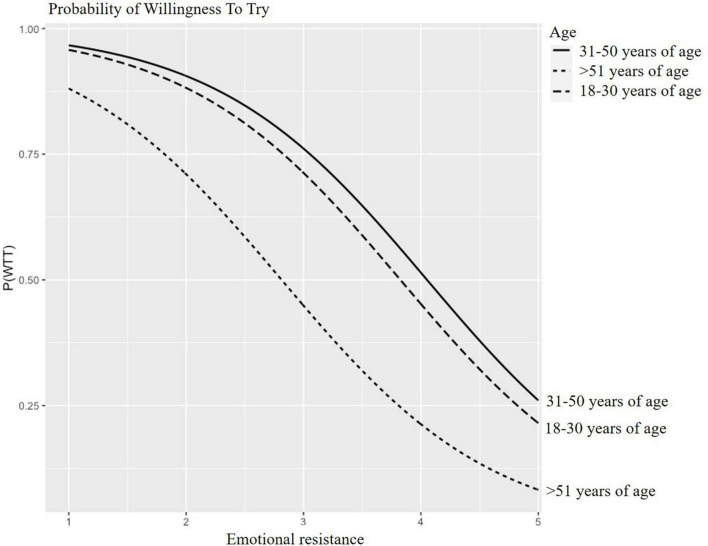
Predicted probability of willing to try (WTT) cultured “meat” (CM) depending on emotional resistance for different age participants.

**FIGURE 3:**
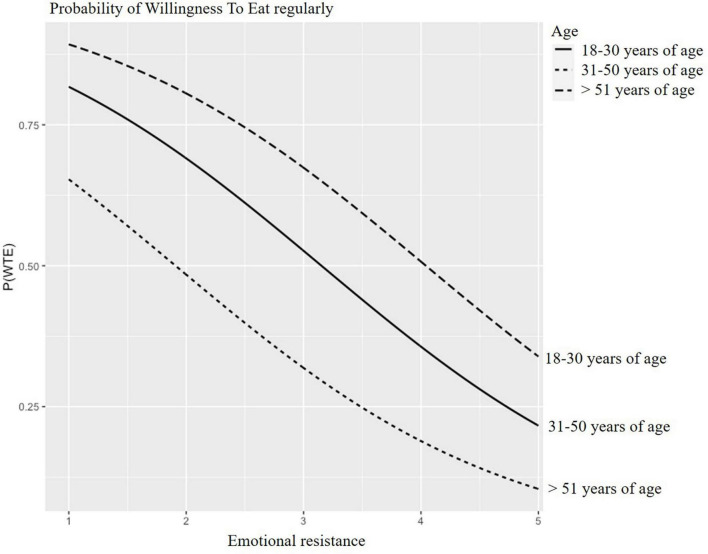
Predicted probability of willingness to eat regularly (WTE) cultured “meat” (CM) depending on emotional resistance for different age participants.

**FIGURE 4:**
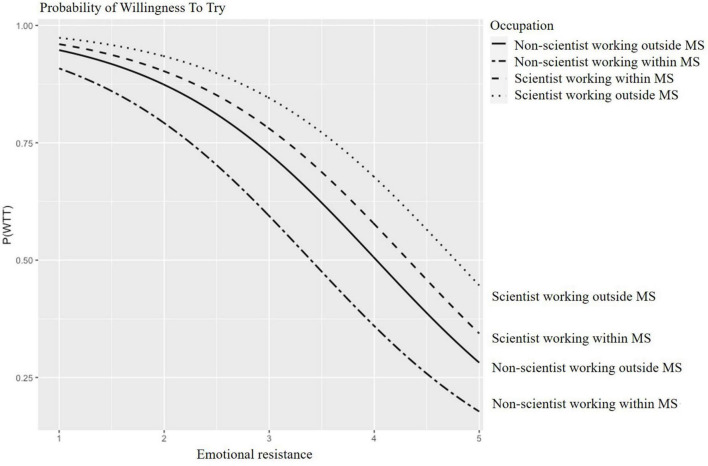
Predicted probability of willing to try (WTT) cultured “meat” (CM) depending on emotional resistance for participants working in different areas. MS, meat sector.

**FIGURE 5:**
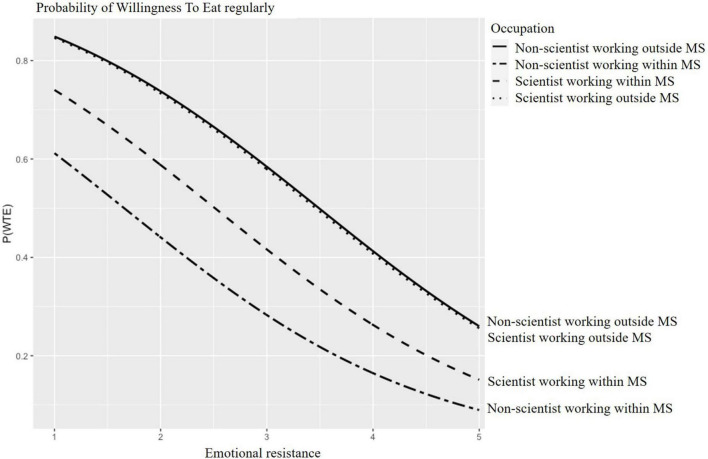
Predicted probability of willingness to eat regularly (WTE) cultured “meat” (CM) depending on emotional resistance for participants working in different areas. MS, meat sector.

Predicted probabilities for WTT and WTE CM are presented in Figures 2–5 for participants of different age groups and different occupations across the range (1–5) of emotional resistance. Negative effects on WTT and WTE can be observed in all figures with increasing level of emotional resistance.

Figure 2 displays the simulated impact of emotional resistance on WTT CM for young, middle-aged and old participants. For an average emotional resistance of 3, the probability of WTT CM amounts to approximately 45% for old participants, 70% for young participants and 75% for middle-aged participants.

Different trends can be seen for WTE (Figure 3). Indeed, for an average emotional resistance of three, the probability of WTE CM amounts to approximately 30% for old participants, 55% for middle-aged participants and 70% for young participants.

These results demonstrate that emotional resistance has the most impact on willingness to try and to regularly eat CM for old people, and has the least impact to try CM for middle-aged people and has the least impact to regularly eat CM for young people.

For a low emotional resistance (value of one), the resulting probabilities of WTT amounted to a maximum of approximately 90% for all the participants working in different areas; assuming emotional resistance at the highest value of five, the resulting probabilities of WTT amounted to a minimum of 10% for people who were not scientists but work within the meat sector and of 45% for people who were scientists work outside the meat sector (Figure 4).

A larger effect of emotional resistance can be seen in Figure 5, according to the work area of respondents. People working outside the meat sector regardless they were scientists or not expressed the same WTE. With those participants, emotional resistance has the least impact on their WTE. Assuming emotional resistance at the lowest value of one, the resulting probabilities of WTE amounted to a maximum of approximately 85% for the participants working outside the meat sector, of 75% for scientists working in the meat sector and of 60% for people who were not scientists but working within the meat sector. Assuming emotional resistance at the highest value of five, the resulting probabilities of WTE amounted to a minimum of 10% for people who were not scientists but work within the meat sector and of 15% for meat scientists and of 25% for those who work outside the meat sector.

## Discussion

### Perceptions and acceptance of CM in different countries

This survey translated into Italian, Spanish and Portuguese and distributed in countries in the South-West of Europe has provided novel results in addition to previous data from France (29), China (27) and Brazil (28). Emotional resistance associated to some food externalities (i.e., disgust, neophobia) is generally cultural dependent (32). Although people with origin from the South of Europe are known to be more conservative than Northern countries, the current respondents from Italy, Spain and Portugal seem to have a quite positive attitude towards CM (49% of the current sample considered CM as “promising and/or acceptable” and 29% perceived CM as “absurd and/or disgusting”) when compared to the French participants (17% for “promising and/or acceptable” and 59% for “absurd and/or disgusting”) (29), but less positive than Chinese and Brazilians, 15 and 18% of their samples perceived CM as “absurd and/or disgusting,” respectively (27, 28; Table 6). With different sample sizes, the same proportion of respondents (33%) from the current samples and Brazilians had higher emotional resistance towards CM, which is expectedly, lower than the French proportion (56%) and higher than the Chinese proportion (16%) (Table 6). The emotional resistance may be associated to perceived immorality of innovation, distrust of new technologies, food neophobia (33), and also concerns about the decline and collapse of tradition (i.e., conventional livestock farming, traditional grazing landscape) (29).

**TABLE 6:** Perceptions and acceptance of cultured “meat” (CM) in different countries.

	Perception			Emotion	Willingness		
Country of origin	Promising and/or acceptable	Fun and/or intriguing	Absurd and/or disgusting	Resistance	WTT	WTE	Higher WTP
Italy, Spain, Portugal	49%	23%	29%	33%	66%	57%	6%
France^1^	17%	24%	59%	56%	51%	20%	8%
China^2^	36%	49%	15%	16%	50%	53%	4%
Brazil^3^	47%	35%	18%	33%	66%	60%	6%

^1^Data from Hocquette et al. (29).

^2^Data from Liu et al. (27).

^3^Data from Chriki et al. (28).

However, even though the majority of current consumers from South-Western Europe have a more positive view of CM, and are more willing to try and eat it (around 66% WTT, 60% WTE), they are not willing to pay a premium for it (only 6% WTP more than conventional meat) as in France, Brazil and China (27–29). This is in line with the conclusion that positive perception is not necessarily predictive of the potential WTP for CM (34). For the time being, consumers still prefer conventional meat for the same price, even if CM is available at a significant discount (35). Although consumers are willing to try CM, when it comes to WTP, most prefer not to consume it (36, 37).

In overall terms, we found that the current respondents from the South-Western Europe have a similar level of acceptance of CM to those in Brazil, which is higher than those in France (50% WTT, 20% WTE, 8% WTP more) and even in China (50% WTT, 53% WTE, 4% WTP more) (Table 6). In general, Europeans are still reluctant to accept CM (38) compared to Americans (23, 39) and Chinese (27, 40). Especially in areas with a strong agricultural tradition such as France, they are particularly concerned about the origin and production process of agri-food (41). Meat alternatives including CM would be considered as ultra-processed food with safety concerns (41). According to the European consumers in the Netherlands, Poland, Spain, and Finland, and also the United Kingdom, CM was the least (6%) accepted protein alternative compared to plant-based (58%), single-cell (20%) and insect-based (9%) protein (42). Although sample sizes and demographics of respondents vary across studies, it seems that consumers from Germany, Italy, Spain and the Netherlands, for example, are more likely to accept CM than those from France and Belgium (29, 34, 43–46). We found that in our study, the current Spanish participants tend to accept CM more than the Italian and Portuguese ones. According to different comparative studies of consumer perception of CM, Spanish consumers had indeed higher trust and acceptance, and lower food neophobia and disgust towards CM than people from other European countries such as France, Germany, Sweden, and even UK and Brazil (47, 48), and it was reported that Spanish consumers would be ready to buy CM if it would be affordable (49). Comparing to the major European countries from the North that are leading the race such as the Netherlands, there are several CM start-ups currently operating in Spain and the Spanish government is also investing in the CM sector (50). These initiatives may boost citizen’s awareness of CM from different angles. By comparison, Italy is where valuable indigenous cattle breeds are largely raised and is the country where the prestigious PDO [Protected Designation of Origin (food and wine)] and PGI [Protected Geographical Indication (food and wine)] are largely located. Italian cuisine has influenced gourmets across Europe and around the World (51). Meat consumption is therefore significant and important in Italy, despite the fact that Italians are increasingly sensitive to the negative effects of the conventional meat sector (51). In 2020, more than half of Italian consumers stated that they would reduce their meat consumption in order to meet the principles of ethical consumption and there are 8% Italians who chose a vegetarian diet and this number is continuously growing (52). The traditional consumption of meat and the influence of emerging trends will have a decisive impact on the acceptance of CM in Italy. Moreover, we found that the current respondents from the South-Western Europe have a similar level of acceptance of CM to those in Brazil, we assume that some common points (i.e., language, culture) between Brazil and Portugal might be able to explain part of the similar acceptance of CM.

### Potential profile of CM adopters and rejectors

According to previous published results, the profiles for potential consumers of CM can be, on average, young and well-educated people, vegetarian, and aware of the technology of CM to some extent (22, 34, 35, 53). Our results confirm these observations since we found that young and higher educated people, also people who are familiar with CM would be more willing to accept CM (summary in Table 7). Notably, we found that people who work outside the meat sector and/or work in academia as scientists would be more willing to try, to eat and to pay more for CM.

**TABLE 7:** Potential profile of cultured “meat” (CM) adopters and rejectors.

Potential profile of CM adopters	Potential profile of CM rejectors
Young	Old
Higher educated	Less educated
Familiar with CM technology	Not familiar with CM technology
Work outside the meat sector	Work within the meat sector
Scientist	Non-scientist

With the current respondents, gender, age, origin and occupation have significant effects on the acceptance of CM. While there is no doubt that people of 18–30 years of age had the highest acceptance of CM, 31–50 years middle-aged people also seem to have a higher willingness to try but not regularly eat compared to young people. Moreover, although there was no difference of WTT and WTE between females and males, we do notice that the possibility of WTT and WTE for males was significantly higher than females. Wilks et al. (54) found that the effect of age and gender are more important for acceptance than education level. This is consistent with our results. Indeed, despite that higher educated people had higher WTT than lower educated people, the predictive effect of education level is not significant in the logistic regression model.

Similarly, with the current respondents, meat consumption level is not a significant factor influencing CM acceptance, but we noticed that the current vegetarians and low meat eaters would be willing to pay more for CM than heavy meat eaters. This is in contrast with the finding that meat consumers rather than vegetarians/vegans seem to be willing to pay more for CM (34). However, it seems that there has always been controversy regarding vegetarian acceptance of CM depending on the motivations to adopt the vegetarian diet. In some studies, vegetarians were more likely to accept CM (23), but in others, vegetarians were less likely to consider the consumption of CM (55), due to concerns about such as healthiness and safety (45). Vegetarians may accept to eat CM to avoid slaughtering animals or may not accept to eat CM, because they refuse to eat any type of meat (including meat from cultured muscle cells). Therefore, it seems difficult to conclude about any effect of meat consumption level on potential CM acceptance.

Although those more familiar with CM had a higher WTT and WTE, the predictive effect of familiarity is not significant, which would suggest that it is unreliable to predict acceptance based merely on familiarity with CM. Nonetheless, we found that people who heard about CM tend to be more likely to try CM but less likely to consume it regularly. This may suggest that regular consumption of CM is unacceptable for consumers at the present stage, even if they are willing to try but maybe just due to curiosity. Anyhow, results from the literature are not consistent in this area: Rolland et al. (44) and Siegrist and Hartmann (48) observed that the previous knowledge of CM can be a good predictor of consumer acceptance, but, however, providing too many technical details to consumers may reduce consumer acceptance (48).

Bryant et al. (43) found that people who work in the sector of animal agriculture or meat production were more likely to accept CM. The associated explanation is that farmers may believe that CM can be an effective means of meeting increasing meat demands and of transitioning away from intensive industrial productions. As the authors mentioned, this might be counter-intuitive. Indeed, we may think that farmers would be opposed to a technology that is likely to replace their own professional activity (56). This can explain why we observed in our study that people working in the meat industry had the least willingness to accept CM. These people have a stronger emotional resistance towards CM. And, even if they have the same level of emotional resistance, those working in the meat sector have a lower WTT and WTE than those not working in the meat sector.

We also found that people working as scientists in academia were more willing to try, eat and pay more for CM. This is logical since people who work in the scientific area are likely to be more open to any technology. They might be also more aware of the principle of CM production, and they may know better the importance of technical expertise and financial investment required for innovation, so they are more willing to pay a premium. Their views on CM are more likely to be rational perceptions based on science and technology as opposed to emotional fear or disgust to something unknown on the one hand or to quick adherence to concepts disseminated on social networks on the other hand. Alternatively, scientists being mainly motivated by science and technology, their opinions regarding social consequences of the development of any technology might be less robust compared to stakeholders and politics.

### The potential motives and barriers of the current acceptance of CM

Cultured “meat” has emerged in a period where the ethical, environmental, safety issues regarding conventional meat production have been subject to growing criticisms. To reach the goals of a sustainable meat production, CM aims at guaranteeing global food security while reducing animal suffering and preserving environmental resources (15). The potential benefits of producing meat *in vitro* have been advocated by CM proponents for a long time, including by some highly influential celebrities (i.e., Bill Gates). Under the influence of these privileged people and due to socially influential activities (such as the protests against animal slaughter and referendums on animal welfare and environmental protection), it may become politically correct to accept CM. In other words, with the constant propaganda about the advantages of CM and the disadvantages of conventional meat, to address environmental, ethical and safety problems that caused by conventional meat production, citizens awareness will be boosted, and they may have to end up by accepting CM. Hence, CM is indeed perceived as a promising new field. This can be seen in the large number of articles that continue to be published in the press media despite a low scientific background (57). Although CM can avoid mass slaughter and exploitation of animals (58), once reliance on fetal bovine serum is no longer necessary, this technology is perceived as ahead of morals. However, the fact that the production process does not fit with the current European law that meat should originate from animal flesh, not from cell culture is also a moral issue. This is also the origin of emotional resistance caused by food neophobia and disgust that has a negative effect on CM acceptance (48). Even leaving aside the different nature of producing meat in a conventional or artificial context, food fraud issue also deserves caution. As demonstrated by Treich (59), CM and conventional meat may become indistinguishable as technology is constantly and rapidly updated. In this way, conventional meat could be fraudulently substituted, which would cause threaten and challenges to consumer welfare and market regulation.

Weinrich et al. (46) found that ethical concern is a strong driver that affects consumer acceptance. In fact, we found that the concern of environment is as strong as ethics to affect consumer WTT and WTE CM (correlation coefficients between ethical, environmental concern and WTT are 0.24 and 0.27; with WTE are 0.37, 0.37, *p* < 0.001, summary in Table 8). As mentioned above, the advantages of CM in terms of animal welfare and environmental protection have been promoted for a long time, despite the latter at least is controversial (14) and need to be considered in depth. According to some authors, as the production of CM would be progressively optimized, much fewer resources (60) and energy might be required and more environmental-friendly and sustainable production could be achieved (61), but this is not clear yet (14). CM also requires no management of carcass waste and may have less transport and refrigeration costs and it is expected that CM should have a longer shelf life than conventional meat (13). However, at present, a large amount of energy is still needed to produce CM (i.e., ingredients producing, bioreactor running and post- processing, etc.). Therefore, the issues involving land use, energy use and carbon opportunity cost and their precise estimation are still key to determine the environmental benefits that CM could contribute at this stage (14, 62). In addition, CM generally produces less emissions than conventional meat, but more than plant-based meat substitutes (63) and it could cause even worse environmental damage in some scenarios. Indeed, emissions from CM production consist mainly of carbon dioxide, which will remain in the atmosphere for longer than methane and nitrous oxide, the main greenhouse gases emitted by conventional meat production (64). It is clear that the CM industry has put focus on a more sustainable production with improved efficiency on cost and resource use (15). As a consequence, the current trend seems to be that public trust is being gradually built up by the support of this innovation, before those environmental benefits are actually fully achieved. That is why it is still necessary to continue to carry out consumer acceptance studies, although it is difficult to anticipate and obtain precise data of future consumer acceptance. It is key to better understand the drivers and barriers of the perception of CM.

**TABLE 8:** Potential motives and barriers of cultured “meat” (CM) acceptance.

Potential motives of CM acceptance	Potential barriers of CM acceptance
Concerns about environmental impacts of livestock	Emotional resistance
Ethical concerns	Lower perception of CM benefits in terms of environmental and ethical impacts
A better understanding of CM technology and less knowledge in meat production	Less knowledge about CM technology and more knowledge in conventional meat production
A better understanding of science and technology in general	Lower education level and less understanding of science and technology
Perception that CM may be tasty, safe and healthy	Perception that CM may be not tasty, safe and healthy

Our research shows that the overall perceptions of conventional meat production and CM have both significant impacts on the acceptance of CM. For the current respondents, the more they consider the conventional meat production causes serious ethical and environmental problems, the more they agree that reducing meat consumption could resolve these problems, the more likely they would be willing to try, to regularly eat and to pay for CM. Likewise, the more they believe CM can be ethical, eco-friendly, tasty and safe, the more likely they would be willing to accept CM. On the contrary, if people are less convinced that CM could be more ethical, eco-friendly, tasty and/or safe than conventional meat, this novel food technology, which is not yet widely available, would provoke higher emotional resistance, which would further result in more reluctance to accept CM, especially for the first attempts to try (since WTT is more correlated with emotional resistance than WTE and WTP). However, this observation is based on the current respondents, which are composed mainly of young and middle-aged people. The respondents of our sample are indeed younger than the actual populations of the studied countries. As it was demonstrated by Mancini and Antonioli (51), today young consumers’ choices, based on more ethical principles, will contribute to shape the future market of meat and also of CM.

Moreover, the significant effects of age and occupation indicate that consumer acceptance of CM is highly affected by these two factors in addition to emotional resistance. According to logistic regression analysis, we do find that people different in age and occupations have different levels of emotional resistance and consequently various acceptance level of CM. Older people and people working in the meat sector, especially grassroots workers (i.e., non-scientists) are more likely to be emotionally resistant to CM and thus refuse to try and eat it. Conversely, young people and those working outside the meat sector, especially scientists, are more likely to be less emotionally resistant and more likely to accept CM.

Therefore, to give a more general conclusion based on the factors covered by our study, the negative impact of conventional meat and the positive impact of CM on issues that concern consumers (namely ethical and environmental issues) can be the motives of acceptance of CM. Conversely, issues for which CM may have weaknesses compared to conventional meat and emotional resistance would be the main barriers to accept CM. However, while these findings may be useful, they may be also biased, at least in part, by the lack of such products on the market and by the way information has been provided to respondents on the potential benefits or drawbacks of both conventional meat and CM. Overall, these findings provide insights into consumer perception and acceptance of CM that can be used by independent academia, and industries of conventional meat and CM. Not only consistent findings but also variabilities in the potential acceptance of CM by different consumer segments are important for the future communication on consumer study of CM. As it is highlighted by Faletar and Cerjak (65), the development and even success of CM in the current marketplace depends firstly on the advancement of the technology and how eco-friendly, ethically and economically the production process can be. However, it also mainly depends on the moral, ethical, economic perception consumers may have about this novel product and on their potential acceptance limited by some emotional resistance.

### Limitation concerning sampling and representativeness

In general, the sample collected for this study consisted of a slightly more female, young, middle-aged and Italian population. It is certainly the most rigorous and correct approach to collect demographically representative data for a questionnaire-type study. Nevertheless, are survey data that are less strictly representative can be effective to convey some useful information? In recent years, there has been a proliferation of research studies on CM, using sample data that are either representative of the local population (43) or not, which seem to be more often not representative (22, 31). However, through our research, as well as previous research and review articles (48), the perceptions and acceptance of CM among consumers with different origins and backgrounds, while not identical, but basically move up and down on the same trends. One of the essences of survey-type questionnaires is to reflect a certain trend through a large sample of data. Undoubtedly, the trend reflected by an extremely biased sample will also be extremely biased, which is definitely not our case. But it cannot be guaranteed that the results demonstrated by a strictly representative sample are completely accurate and representative. One of the purposes of this study is to reflect the overall consumer perception and acceptance of CM in three countries from South-Western Europe. Despite for the limitation of sampling and representativeness, comparing results obtained with the same experimental design between countries or between similar consumer segments could provide useful information.

## Conclusion

Consumer acceptance is critical for the success of the CM industry. This study sheds light on how consumers from Italy, Spain, and Portugal perceive CM and their acceptance. In comparison with the previous data, the current Italian-, Spanish-, and Portuguese-speaking and/or originated people seem to have a more positive attitude towards CM especially compared with French samples. About a quarter of people have a negative view (absurd and/or disgusting) or emotional resistance towards CM. According to the current participants, the acceptance of CM tends to be higher for 18–30 years-old people and for respondents who work outside the meat sector especially scientists, and people who already heard about CM and with a higher acceptance for males. By comparison with respondents originated from Italy and Portugal, Spanish respondents seem also to have a higher propensity to accept CM. The high predictive effects of age and especially occupation indicate that these two factors can be good indicators of consumer acceptance of CM, which tends to be larger among young people and people working outside the meat sector.

Issues arising from conventional meat production that can be addressed by CM, for example with regard to animal welfare and the environment, can be among the major motives for the current respondents to try and regularly eat CM. For instance, according to proponents of the CM industry, the perceptions that CM may be more eco-friendly, ethical and healthy than conventional meat could motivate consumers to consume CM. On the opposite, price, the emotional resistance induced by CM and the negative impacts of CM for consumers (in terms of safety and healthiness for example) would be the barriers for the current respondents to accept CM.

## Data availability statement

The raw data supporting the conclusions of this article will be made available by the authors, without undue reservation.

## Ethics statement

Ethical review and approval was not required for the study on human participants in accordance with the local legislation and institutional requirements. The patients/participants provided their written informed consent to participate in this study.

## Author contributions

ÉH, JL, M-PE-O, SC, and J-FH: conceptualization. JA, NR, and BP: data collection. JL: curation, formal analysis, and writing—original draft. JL and J-FH: investigation and methodology. M-PE-O, SC, and J-FH: supervision. JL, M-PE-O, SC, and J-FH: validation. JL, JA, NR, BP, ÉH, SC, M-PE-O, and J-FH: writing—review and editing. All authors read and agreed to the published version of the manuscript.
